# Assessing the efficacy of herbal supplements for managing obesity: A comprehensive review of global clinical trials

**DOI:** 10.22038/ijbms.2025.84150.18198

**Published:** 2025

**Authors:** Mahboobeh Ghasemzadeh Rahbardar, Gordon A Ferns, Majid Ghayour Mobarhan

**Affiliations:** 1 Clinical Research Development Unit, Shahid Hasheminejad Hospital, Mashhad University of Medical Sciences, Mashhad,Iran; 2 Brighton and Sussex Medical School, Division of Medical Education, Falmer, Brighton BN1 9PH, Sussex, UK; 3 Department of Nutrition, Faculty of Medicine, Mashhad University of Medical Sciences, Mashhad, Iran; 4 Iranian UNESCO Center of Excellence for Human Nutrition, Mashhad University of Medical Sciences, Mashhad, Iran; 5 Metabolic Syndrome Research Center, Mashhad University of Medical Sciences, Mashhad, Iran

**Keywords:** Appetite regulation, Body mass index, Body weight, Herbal medicine, Obesity management, Plant extracts, Waist circumference, Weight loss

## Abstract

Obesity remains a significant worldwide health concern, and further research into other strategies, including herbal weight-loss medications, is necessary. By reviewing clinical trials, this study aims to evaluate the effectiveness of herbal medicines for weight loss or obesity. A comprehensive search was conducted using multiple databases. Clinical trials evaluating the effects of herbal medicines on weight loss or obesity management were included. Relevant data, such as study design, intervention details, and outcome measures, were extracted and analyzed. The use of herbal medicines exhibited varying efficacy in promoting weight loss or managing obesity. Some herbal interventions significantly reduced body weight, body mass index (BMI), and waist circumference. Notably, these interventions led to decreases in fasting blood glucose (FBG) and homeostatic model assessment of insulin resistance (HOMA-IR), regulating insulin levels while increasing levels of catalase (CAT) and glutathione (GSH). Additionally, reductions in inflammatory markers such as high-sensitivity C-reactive protein (hs-CRP) and tumor necrosis factor-alpha (TNF-α) were observed, indicating a potential anti-inflammatory effect. Mechanisms of action included appetite regulation, fat oxidation, increased satiety, enhanced insulin sensitivity, and modulation of lipid metabolism. However, it is important to note that these herbal interventions’ efficacy and safety profiles may vary among different population groups. The findings suggest that certain herbal medicines hold promise as adjunctive therapies for weight loss and obesity management. However, comprehensive and targeted research efforts are warranted to determine these herbal interventions’ optimal use, dosages, and long-term effects in specific population subgroups.

## Introduction

Obesity is characterized by accumulation of excess fat in the body due to an imbalance between energy intake and expenditure. Obesity is becoming more prevalent globally due to changes in human lifestyle (1). It is often associated with a poorer quality of life. It is a major risk factor for a variety of illnesses, including dyslipidemia (2-4), hypertension (5), type 2 diabetes (6), osteoarthritis (7), sleep disturbances, some malignancies (1), metabolic (8, 9), cardiovascular (10), respiratory diseases (6), and renal diseases (1). Obesity can also increase the risk of premature mortality (11). Despite an abundance of research on obesity treatment and management, the global incidence of this disorder remains a challenging issue (1).

The pathophysiology of obesity is a complicated process affected by several genetic, environmental, and behavioral variables (12). Environmental factors, such as sedentary lifestyles, play an important role. Furthermore, a poor diet, such as eating high-calorie foods, is a key contributor to obesity (13, 14).

Understanding the underlying mechanisms of obesity is crucial in developing effective interventions (15). Adipose tissue, previously considered only an energy storage center, is now recognized as an active endocrine organ that regulates metabolic homeostasis (16). Adipocytes produce and release various bioactive molecules, including adipokines and cytokines that control appetite, energy expenditure, and insulin sensitivity. Obesity and its consequences can result from dysregulation of these signaling pathways (17).

Typical pharmaceutical therapies for obesity, such as appetite suppressants or nutrition absorption inhibitors, have been developed over time. However, they may have side effects. For example, pharmaceutical medications that target appetite frequently result in slight weight loss and may have side effects such as elevated heart rate and psychological symptoms (18). These limitations have led to a growing interest in investigating alternative approaches to weight management.

In recent years, herbal medicines have garnered increased attention as promising interventions across various health fields (19-22). Herbal medicines derived from plants have been used in traditional medicine for centuries and could effectively prevent obesity. These natural substances contain various bioactive ingredients that can influence metabolic pathways related to energy balance, adipogenesis, lipid metabolism, and appetite regulation (5, 11). However, it is important to note that the natural origin of these substances does not inherently imply superiority or absence of potential side effects. Understanding these herbal treatments’ pharmacological mechanisms can provide significant information about their potential efficacy and safety as anti-obesity medicinal products.

Thus, this review aims to explore the published research and investigate the potential of herbal medications as a therapeutic option for managing the global obesity epidemic. By investigating the underlying pharmacological mechanisms and evaluating clinical trial evidence, we aimed to shed light on these herbal treatments’ efficacy, safety, and therapeutic potential. Discovering the effects of herbal medicines in weight management may help develop novel strategies for treating obesity and its related health problems.

## Methods

Our team employed a structured approach to synthesize and analyze the available literature on the subject to perform a comprehensive and transparent evaluation. Our methodology included the following steps:

1. Literature search strategy: A thorough search across multiple databases was undertaken, including Google Scholar, PubMed, and Scopus, utilizing a comprehensive set of search terms related to weight loss, obesity, herbal medicine, and complementary medicine. The used keywords comprised “alternative medicine”, “anti-obesity”, “botanical”, “botany”, “complementary medicine”, “ethnomedicine”, “herbal drugs”, “herbal medicine”, “herbal preparations”, “herbal product”, “herbal remedy”, “herbal”, “herbs”, “medicinal plant”, “natural product”, “naturopathy”, “obesity”, “pharmacognosy”, “phytochemical”, “phytocompound”, “phytomedicine”, “phytotherapy”, “plants medicinal”, “slimming”, “traditional medicine”, “Unani medicine”, and “weight loss”. 

2. Inclusion criteria: We included clinical trials that evaluated the effects of herbal medicines on weight loss or the management of obesity. Studies meeting the predefined criteria were considered for analysis.

3. Search duration: The literature search was conducted without any time restrictions up to June 2024 to ensure comprehensive inclusion of relevant studies published until that date.

4. Data extraction and analysis: Relevant data, such as study design, intervention details, and outcome, were extracted to assess the efficacy of herbal medicines in promoting weight loss and managing obesity.

## Obesity and underlying mechanisms

Obesity, defined as the excessive accumulation of body fat, has far-reaching health consequences and is related to a variety of co-morbidities (23). Understanding the underlying mechanisms is critical for understanding the implications of obesity and developing efficient treatments.

Oxidative stress is associated with obesity. Excess body fat alters the balance between pro-oxidants and anti-oxidants, resulting in elevated levels of reactive oxygen species (ROS). Oxidative stress damages cells and disrupts normal metabolic processes. Malondialdehyde (MDA) is a marker of oxidative stress that assesses lipid peroxidation, whereas anti-oxidants such as glutathione (GSH), superoxide dismutase (SOD), and catalase (CAT) protect against oxidative damage. Obesity increases MDA levels while decreasing GSH, SOD, and CAT activities, which contributes to cellular damage and dysfunction (24, 25). Furthermore, oxidative stress activates nuclear factor-kappa B (NF-κB), a transcription factor that causes inflammation (26).

**Figure 1:**
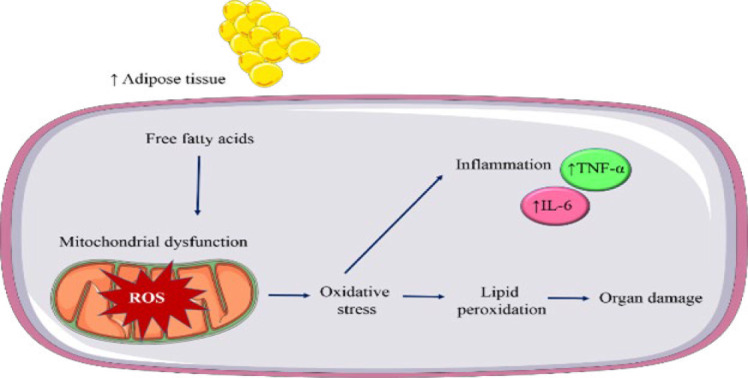
Suggested role of oxidative stress and inflammation in obesity (Images from https://smart.servier.com)

Inflammation is another important consequence of obesity. Excess adipose tissue is infiltrated with immune cells, particularly macrophages. These immune cells release pro-inflammatory cytokines, including tumor necrosis factor-alpha (TNF-α) and interleukin-6 (IL-6). These cytokines increase insulin resistance, interfere with adipocyte function, and cause metabolic disorders. Obesity causes persistent low-grade inflammation, contributing to oxidative stress and cardiovascular and metabolic complications (27) (Figure 1).

Liver enzymes, such as alanine aminotransferase (ALT), aspartate aminotransferase (AST), and gamma-glutamyl transferase (GGT), provide insights into liver function (28). Elevated ALT and AST levels indicate liver damage, which is frequently associated with nonalcoholic fatty liver disease (NAFLD), a prevalent obesity-related disorder (29). Increased GGT levels are associated with oxidative stress and liver dysfunction (30), emphasizing the complex relationship between oxidative stress, inflammation, and liver health in obesity.

Hormonal factors also contribute to obesity (31, 32). Leptin, an adipokine produced predominantly by adipose tissue, regulates hunger and energy balance by signaling satiety to the brain (33). Some individuals with obesity develop leptin resistance, which impairs appetite regulation and energy balance (34). Adiponectin, another adipokine, possesses anti-inflammatory and insulin-sensitizing properties (35). Reduced adiponectin levels in obesity may contribute to insulin resistance and metabolic dysfunction (36). Ghrelin, which is primarily produced in the stomach, increases appetite and encourages food consumption. Its dysfunction in obesity may lead to increased calorie consumption (37).

**Figure 2:**
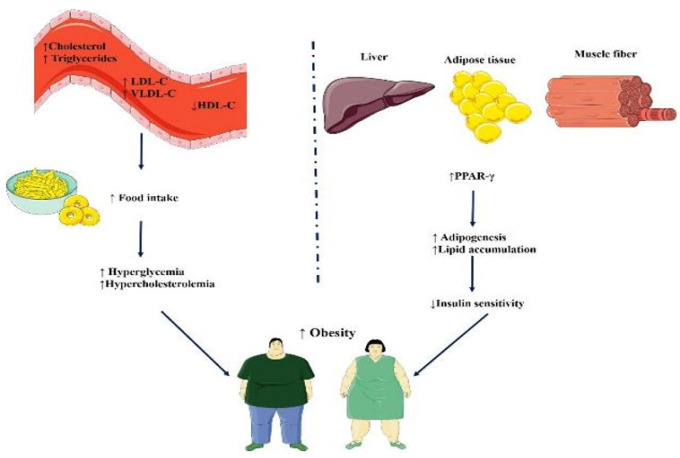
Proposed role of hyperlipidemia, insulin sensitivity, and PPAR-γ in obesity (Images from https://smart.servier.com)

Obesity might also be associated with changes in serum lipid profiles (Figure 2), such as increased low-density lipoprotein (LDL) and triglycerides (TG) and decreased high-density lipoprotein (HDL). These lipid abnormalities increase the risk of cardiovascular disease and metabolic complications (38).

Other factors associated with obesity include elevated glycosylated hemoglobin (HbA1C) levels (indicative of long-term blood glucose control) (39), increased insulin resistance as assessed by homeostatic model assessment of insulin resistance (HOMA-IR) (40), elevated high-sensitivity C-reactive protein (hs-CRP) levels (a marker of systemic inflammation) (41), upregulated intercellular adhesion molecule-1 (ICAM-1) expression (involved in immune cell recruitment) (42), and dysregulation of transcription factors such as peroxisome proliferator-activated receptor-gamma (PPAR-γ) (43), and sirtuin 1 (SIRT1) (44), which play roles in adipogenesis, inflammation, and metabolism.

Examining the interrelationships among these fundamental mechanisms can help achieve a comprehensive understanding of the complex nature of obesity and its associated detrimental effects. By focusing on and modifying these pathways, interventions can be developed to lessen the adverse effects of obesity while improving general health outcomes.

## Herbal medicines with reported anti-obesity effects

*Allium sativum*

One of the most significant and historically documented plants is garlic (*Allium sativum* L.; Family: Amaryllidaceae), an aromatic annual spice used in traditional medicine since ancient times (45, 46). Based on epidemiological data from clinical studies, *Allium* species, and their active components have been shown to lower the risk of diabetes and cardiovascular diseases, protect against infections by boosting the immune system, and have anti-microbial, anti-fungal, anti-aging, and anti-cancer properties (47, 48).

It has been reported that dietary supplementation of *A. sativum* powder in patients with NAFLD led to reduced body weight and body fat mass (49). However, it did not alter lean body mass and total body water (49). Moreover, it has been found that consuming *A. sativum* powder could enhance muscle mass and attenuate body fat and visceral fat levels in postmenopausal women compared to their pre-test data (50).

The findings of a clinical trial on NAFLD patients demonstrated that administration of *A. sativum* powder caused an increase in skeletal muscle mass, serum levels of SOD, and total anti-oxidant capacity (51). Its use also decreased waist circumference, body fat percent, fasting blood glucose (FBG), insulin, homeostasis assessment model for insulin resistance (HOMA-IR), as well as MDA compared to the control group (51). Furthermore, Allium-S tablets containing *A. sativum* extract lowered body mass index (BMI), fasting insulin level, and HOMA-IR in obese women. It also modified gut microbiota by augmenting *Faecalibacterium* and *Bifidobacterium* abundance and declining *Akkermansia* frequency (52). Similarly, *A. sativum *substantially lowered weight, BMI, and waist circumference in women with polycystic ovary syndrome and enhanced serum CAT and GSH levels (53) (Table 1). 

While these findings suggest that *A. sativum* supplementation may hold therapeutic potential for managing conditions such as NAFLD, obesity, and metabolic disorders, a critical evaluation of the studies is essential. Many studies employed small sample sizes and short durations, limiting the generalizability of the findings; thus, more extensive, long-term studies are needed to confirm efficacy and safety. Variability in methodologies, such as differences in dosages, forms of garlic, and participant characteristics, can also affect outcomes, highlighting the need for standardization. Furthermore, the sustainability of the observed effects over time remains uncertain, necessitating longitudinal studies to determine whether the benefits of *A. sativum* supplementation are maintained after discontinuation. While some studies have suggested mechanisms through which *A. sativum* may exert its effects, such as anti-oxidant activity and modulation of gut microbiota, further research is needed to elucidate these mechanisms and understand their contributions to weight management and metabolic health. 

Regarding the observed increase in muscle mass, potential mechanisms for this effect might include the anabolic properties of *A. sativum*, which may stimulate muscle protein synthesis and promote muscle growth. Additionally, the anti-oxidant properties of *A. sativum* may provide a more favorable environment for muscle growth by reducing oxidative stress and inflammation. Increased nitric oxide production and potential hormonal regulation by *A. sativum* could also contribute to enhanced muscle mass. It is worth noting that these suggested mechanisms are speculative and require further investigation to validate their role in the observed effects of *A. sativum* supplementation. 

*Camellia sinensis*

Green tea, which is derived from the tea plant *Camellia sinensis*, is one of the most popular drinks in Asia (54). *C. sinensis* is composed of various bioactive components such as caffeine, flavonol glycosides, polyphenolic compounds (catechins and epicatechins), L-theanine, theaflavins, theobromine, and volatile organic substances. These constituents contribute to this herb’s distinctive aroma, astringency, flavor, and taste while providing health benefits (55). These bioactive compounds are responsible for the positive effects of tea on human health. Anti-oxidant (56), anti-inflammatory (57), anti-viral (58), anti-bacterial (59), neuroprotective (60), and anti-angiogenic (61) effects are some of the physiological benefits of green tea. This plant also shows anti-obesity advantages, which will be explained in the following section.

Taking Monoselect Camellia® (MonCam), comprising bioavailable *C. sinensis* extract, increased weight loss and lessened BMI, waistline (just in males), as well as leptin in obese individuals (62). Besides, Wang et al. demonstrated that consuming *C. sinensis* with high catechin could attenuate intra-abdominal fat, waist circumference, body weight, and total body fat in moderately overweight Chinese participants (63).

However, the supplementation of epigallocatechin-3-O-gallate, a catechin compound of *C. sinensis*, in obese premenopausal Caucasian women with BMI 30-40 kg/m^2^ had no significant effect on body weight, fat mass, energy, and fat metabolism, HOMA-IR, total cholesterol (TC), LDL, TG, and liver function markers (64). It has been reported that consuming *C. sinensis *had no significant effects on fecal energy content, fecal fat content, resting energy expenditure, respiratory quotient, and body composition on healthy, obese/overweight (BMI >25 kg/m^2^), or normal-weight BMI (18–25 kg/m^2^) Caucasian people (65). 

The administration of a water extract from *C. sinensis *leaf to women with central obesity resulted in increased levels of adiponectin and reduced levels of TC, LDL, and ghrelin. It also decreased weight and BMI (66) (Table 1). Likewise, consuming *C. sinensis* in people with a BMI of 24-35 kg/m^2^ caused a decline in weight and BMI compared to the placebo group (67). A combination of epigallocatechin-3-O-gallate and *Citrus* polyphenol α-glucosyl hesperidin was evaluated for its potential to reduce obesity. Healthy Japanese men and women drank green tea containing epigallocatechin-3-O-gallate along with α-glucosyl hesperidin. By week 12, it lowered BMI and stopped weight gain compared to the placebo. Body weight, BMI, and the blood LDL/HDL ratio all dropped along with the TG and body fat percentage at week six and the visceral fat level and body fat percentage at week 12 (54).

In brief, studies have shown conflicting results regarding the effects of *C. sinensis *extract on weight loss and obesity-related parameters. The contradictory results may be influenced by various factors, including differences in participant demographics and genetic variations among different populations, as well as study design, dosages, and measurement methods. It is worth noting that studies involving Caucasian individuals did not yield significant results. Therefore, considering the inclusion of diverse populations, including different ethnic groups and tribes, in future research may provide valuable insights into the efficacy of *C. sinensis* for weight management and obesity-related parameters. 

*Cinnamomum verum*

There is a long history of using cinnamon in food and medicine. It comes from the stem of the Lauraceae tree, *Cinnamomum verum *J. Presl (syn*. Cinnamomum zeylanicum *Blume). Ancient civilizations in China and Egypt used it as a flavoring ingredient and in traditional medicine. Anti-oxidant, anti-bacterial, anti-inflammatory, and fever-reducing properties are well-known for cinnamon. Furthermore, it has been used to repair tissue and treat several diseases, including bloating, colds, cardiovascular disorders, cramps, diarrhea, gastrointestinal issues, intestinal spasms, nausea, and sore throats (68, 69).

It has been reported that administering *C. verum *to people with type-2 diabetes reduced FBG, HbA1C, TG, weight, BMI, and body fat mass compared to the baseline data (70). The findings of a clinical trial indicated that the administration of *C. verum *to people with type-2 diabetes could successfully lower their weight, waist circumference, and BMI (69). It has been shown that cinnamon increased HDL and reduced FBG, HOMA-IR, TC, LDL, and weight in overweight/obese participants with polycystic ovary syndrome compared to the placebo group. It also reduced TG and BMI compared to the baseline data (71). 

Supplementation of* C. verum* in obese individuals with type-2 diabetes resulted in enhanced ghrelin secretion, decreased BMI and waist circumference, and lowered levels of blood glucose and insulin (72). In the same way, taking *C. verum* in women with polycystic ovary syndrome declined weight, BMI, HOMA-IR, and testosterone levels (73) (Table 2).

Moreover, it has been shown that administration of *C. verum* bark powder to migraine patients prevented the increase in body weight and BMI, whereas the placebo group experienced it. Also, it reduced hip circumference and headache daily, compared to the placebo group (74). Furthermore, *C. verum* and inulin significantly decreased weight, BMI, serum insulin, HOMA index, lipaemic pattern, TC, TG, and LDL in patients with metabolic syndrome and type-2 diabetes or impaired glucose tolerance (75).

Hence, the administration of *C. verum* has demonstrated beneficial effects in various health conditions, particularly in individuals with type-2 diabetes, polycystic ovary syndrome, and migraines. It has shown potential in reducing FBG, HbA1C, TG, weight, BMI, body fat mass, waist circumference, and insulin resistance. Moreover, *C. verum* supplementation has been associated with improved lipid profiles, increased HDL, and decreased testosterone levels. These findings highlight the potential of *C. verum* as a natural therapeutic option for managing diabetes, obesity, and related metabolic disorders. 

*Crocus sativus*

*Crocus sativus* L., also known as saffron, is a valuable medicinal herb. It belongs to the Iridaceae family and has been grown for over 3000 years in certain parts of the world. *C. sativus* stigmas contain components that are predominantly composed of three major metabolites. First, there are crocins responsible for the distinctive *C. sativus* color. Second, picrocrocin, which contributes to the bitter flavor of *C. sativus*. Finally, safranal is responsible for the specific aroma of *C. sativus* (76).

The investigations revealed different advantageous effects of this herb, including anti-apoptotic (77, 78), anti-inflammatory (79), anti-oxidant (80, 81), cytoprotective (82), hypnotic (83), renoprotective (84, 85), neuroprotective (86), anti-depressant (87), and anti-asthmatic (88, 89) properties. 

Examining the effects of Satiereal, a *C. sativus* extract, on healthy, mildly overweight women indicated that consuming Satiereal reduced snacking and had a satiating impact, which may help with weight loss (90). The administration of *C. sativus* aqueous extract and crocin to patients with coronary artery disease revealed that anthropometric measurements and specific body composition indicators in the treatment group showed a pattern of improvement following the intervention. The *C. sativus* aqueous extract group had a considerably more significant decrease in BMI, waist circumference, and fat mass values than the crocin group. The mean energy and dietary intake values were substantially decreased by both *C. sativus* aqueous extract and crocin but were not altered in the placebo group. Additionally, appetite was significantly reduced in the *C. sativus* aqueous extract and crocin groups (91). In contrast, administration of crocin to individuals with metabolic syndrome had no considerable effect on anthropometric measures (92). Another study evaluated the effect of *C. sativus* on overweight/obese prediabetic individuals, showing that although it lowered FBG and HbA1C, it had no effect on anthropometric measures (93). The supplementation of *C. sativus *to patients with type-2 diabetes could significantly decrease waist circumference; however, it showed no significant changes in other anthropometric measures (94) (Table 2).

It has been observed that administration of *C. sativus* to NAFLD patients significantly decreased leptin and hs-CRP levels compared to the placebo group. However, it did not significantly affect patients’ weight, BMI, or visceral fat level (95). Another clinical trial evaluated the effect of *C. sativus* on overweight women with mild to moderate depression. The findings illustrated that *C. sativus* did not significantly affect their food craving. However, subjects who took *C. sativus* continued to lose weight one month after stopping the medication, while those who took a placebo gained weight (96).

*C. sativus* caused no significant alterations in anthropometric measures of patients with mild to moderate ulcerative colitis (97). However, the supplementation of *C. sativus* stigma powder in obese prediabetic adolescents decreased BMI, waist circumference, weight z-score, BMI z-score, TG, and HDL (76).

Despite the mixed results observed across the studies, there was a tendency suggesting that *C. sativus* extract, in various forms such as satiereal or aqueous extract, may positively impact weight loss (90, 91). These contradictory findings among the studies may be attributed to various factors, including differences in study populations, intervention protocols, and measurement methods. These conflicting outcomes highlight the need for further research to fully understand the potential of *C. sativus* extract in weight loss and to identify the specific populations that may benefit the most from its use. It is important to consider the strengths and limitations of these studies. The strengths include randomized controlled designs, comprehensive assessment of various parameters, and the inclusion of placebo groups. However, limitations such as small sample sizes, variations in intervention durations, and the specific contexts of the study populations may affect the generalizability of the results.

*Curcuma longa*

Turmeric, scientifically known as *C. longa*, belongs to the Zingiberaceae family and is a well-known spice. The main active ingredient in *C. longa*, curcumin, provides the plant’s characteristic yellow color. Both *C. longa* and curcumin have diverse physiological properties, including anti-inflammatory, anti-nociceptive (98), anti-oxidant **(**99**)**, anti-apoptotic, and anti-dotal effects (100). They also have anti-obesity properties, which will be covered in the following section.

The effects of curcumin on the anxiety and depression levels of obese individuals were assessed in one study (101). It was observed that although curcumin decreased anxiety scores, it had no significant effect on participants’ weight and BMI (101).

It has been reported that supplementation of curcumin in patients with advanced pancreatic cancer decreased their body weight and subcutaneous fat. It also increased the rate of muscle loss in these patients and finally led to decreased survival rate compared to the untreated group (102) (Table 3). 

Curcumin consumption in NAFLD patients caused a considerable reduction in their liver fat content, BMI, TC, LDL, TG, AST, ALT, serum glucose, and HbA1C (103). Compared to the placebo group, curcumin consumption could successfully decrease weight, BMI, waist circumference, and FBG in overweight samples with type-2 diabetes (104).

A clinical trial was designed to compare the effects of *C. longa* powdered rhizome with/without aerobic training on women with hyperlipidemia and type-2 diabetes. The results showed that body weight, BMI, body fat percentage, and waist-to-hip ratio decreased in all intervention groups. However, the combination of consuming *C. longa* powdered rhizome with aerobic training had more pronounced effects (105). Some overweight 50 to 69-year-old healthy subjects consumed supercritical carbon dioxide extract of *C. longa*, which led to attenuated body weight, BMI, and serum CRP. It also improved their mental health (106).

The findings of a clinical trial demonstrated that administration of curcumin to Brazilian women with high waist circumferences led to lowered body mass, FBG, TG, and TC (107). Taking calebin A, a minor bioactive phytochemical from *C. longa*, resulted in increased HDL, reduced LDL, TG, CRP, and leptin. It also significantly attenuated body weight, BMI, as well as waist circumference in obese patients (108).

It is important to note that the studies have shown positive outcomes in improving metabolic parameters, including liver fat content, cholesterol levels, and glycemic control. These findings suggest that *C. longa* and curcumin may support overall metabolic health and potentially aid weight management. 

On the other hand, the adverse effects of curcumin on pancreatic cancer patients are concerning (102). This highlights the importance of considering the potential negative impacts of *C. longa* and curcumin in specific health conditions, emphasizing the need for caution and further investigation in the field of pancreatic cancer. 

Future research should clarify the mechanisms underlying the anti-obesity effects of *C. longa* and curcumin and identify the specific factors that influence their efficacy. Standardized protocols, larger sample sizes, and longer intervention durations would provide more strong evidence regarding turmeric and curcumin supplementation’s potential benefits and risks for weight loss. Furthermore, exploring the synergistic effects of *C. longa* or curcumin with lifestyle interventions, such as exercise and dietary modifications, could yield more significant and sustainable weight loss outcomes. 

*Garcinia mangostana*

Mangosteen, also known as *Garcinia mangostana* Linn., is a tropical evergreen tree native to Southeast Asia and a member of the *Clusiaceae* family. It usually grows 6-25 meters high and has dark-brown or practically black bark. The fruit of *G. mangostana*, known as “the queen of fruits,” is reddish or dark purple and is prized for its juicy, soft, edible pulp and delicious flavor. In traditional medicine, the pericarp of the mangosteen fruit has been used to treat various illnesses, including chronic ulcers, convulsions, diarrhea, dysentery, fever, infected wounds, pain, stomach discomfort, trauma, and suppuration (109). Studies show mangosteen and its components have significant therapeutic and pharmacological potential. These include anti-oxidant, anti-inflammatory, anti-nociceptive (110), cardioprotective (111), neuroprotective (112), anti-metabolic syndrome (8), anti-malarial and anti-microbial (113), anti-obesity (114), and anti-dotal properties (109).

Consuming XanGo Juice™ (containing the whole fruit puree of *G. mangostana* as a primary ingredient besides other fruit juices) has been reported to decrease hs-CRP and BMI in obese people (115). However, it has been observed that administering *G. mangostana*-based drinks to healthy people had no significant effects on their weight and BMI. However, it increased blood anti-oxidant capacity compared to the placebo group and declined CRP levels compared to the pre-intervention data (116). The results of another clinical trial demonstrated that obese females with a BMI ≥ 30 kg/m2 and body weight less than 135 kg who took a *G. mangostana* supplement in the mangosteen arm experienced weight loss, while the control group did not. Also, a substantial improvement in insulin sensitivity was observed in the *G. mangostana* group (117) (Table 3).

The reviewed studies provide valuable information about the potential effects of consuming *G. mangostana* for weight management. One notable strength of these trials is their inclusion of diverse populations, including both obese individuals and healthy subjects, which enhances the generalizability of the findings. It is worth noting that the administration of *G. mangostana*-based drinks to healthy individuals did not significantly affect weight and BMI. However, it did exhibit an increase in blood anti-oxidant capacity compared to the placebo group and a decrease in CRP levels compared to the pre-intervention data. These findings suggest potential anti-oxidant and anti-inflammatory properties of *G. mangostana*, although its impact on weight management in healthy individuals may be limited. Notably, the plant did show a significant improvement in insulin sensitivity, indicating that this could be a key mechanism underlying its potential anti-obesity effect. 

*Nigella sativa*

*Nigella sativa* is a seed from the Ranunculaceae family, also called black cumin or black seed. It is used as a food preservative and seasoning. *N. sativa* has been used as a natural therapy for a number of ailments, such as depression, rheumatoid arthritis, heart damage, neurotoxicity, hepatotoxicity, and renal toxicity. Thymoquinone, carvacrol, alpha-hederin, thymohydroquinone, thymol, dithymoquinone, nigellicine, nigellidine, and nigellimine-N-oxide are the main active ingredients in *N. sativa* (79).

These active constituents in both *N. sativa* seeds and its oil exhibit pharmacological effects, particularly anti-inflammatory, anti-oxidant, renoprotective (118), anti-hypertensive, anti-hyperlipidemic, and hepatoprotective (16) properties. Moreover, several studies indicated its anti-obesity effects.

Consuming *N. sativa* oil has been reported to reduce BMI, HbA1C, FBG, and two-hour postprandial glucose levels in type-2 diabetic individuals compared to placebo participants (119). Another clinical trial evaluated the effect of *N. sativa *crushed seeds plus an aerobic training program on sedentary, overweight women three times a week. The findings indicated that HDL was increased, and TC, TG, LDL, and BMI decreased in the participants (120). The supplementation of *N. sativa* oil plus a low-calorie diet in obese women augmented the superoxide dismutase (SOD) levels in red blood cells and also decreased their weight in comparison to the placebo group (121).

Similarly, having a low-calorie diet along with receiving *N. sativa* oil pointedly lowered weight, waist circumference, TG, as well as LDL levels in obese women (122) (Table 4). Moreover, it has been reported that the administration of *N. sativa* oil to individuals with type-2 diabetes led to decreased BMI and body weight compared to the baseline. It also declined HbA1C, FBG, TG, and LDL levels compared to the placebo (123). Furthermore, a low-calorie diet along with *N. sativa* oil caused a significant reduction in weight and serum levels of TNF-α and hs-CRP. However, it did not significantly affect BMI and serum IL-6 versus the placebo group (124). Another similar study examined the effect of *N. sativa* oil plus a low-calorie diet on obese women. The data revealed that adiponectin amounts increased body fat mass and insulin levels decreased in samples in the intervention group compared to the placebo group (125). An investigation examined the effect of *N. sativa* oil and calorie restriction on insulin resistance in obese women with PPAR-γ 2 gene polymorphisms. Participants were randomly assigned to one of the two groups: the intervention group, which received *N. sativa* oil, or the placebo group. The comparison of the two groups demonstrated a considerable reduction in body weight in the *N. sativa* oil group, regardless of genotype. However, substantial insulin concentrations and resistance variations existed between individuals with the Pro/Ala and Pro/Pro polymorphisms in the *N. sativa* oil groups. When paired with calorie restriction, *N. sativa* oil lowered weight regardless of genotype; however, its effect on insulin concentrations and insulin resistance was modified by the Pro/Ala genotype (126). 

The administration of *N. sativa* powdered seeds to samples with Hashimoto’s thyroiditis could significantly decrease BMI, body weight, LDL, and TG levels compared to the placebo group (127). A daily dose of *N. sativa* oil was found to be less efficient than metformin in lowering FBG, two-hour postprandial glucose levels, and HbA1C levels in patients recently diagnosed with type-2 diabetes while also enhancing β-cell function. On the other hand, *N. sativa* oil showed comparable efficacy to metformin in markedly lowering BMI, waist circumference, and weight. Furthermore, the effects of *N. sativa* oil and metformin on insulin sensitivity, insulin resistance, insulin sensitivity during fasting, ALT, HDL, LDL, TC, TG, and total anti-oxidant capacity were similar. On the other hand, metformin caused AST and creatinine levels to rise significantly, but the *N. sativa* oil group did not experience these changes (128). 

It has been shown that the administration of *N. sativa* oil to obese or overweight healthy women caused a reduction in BMI, body weight, waist circumference, body fat mass, body fat percent, and visceral fat area compared to the placebo group (129). Assessing the effect of *N. sativa* oil on obese prediabetic samples demonstrated that *N. sativa* oil had similar statistical effects to metformin in improving anthropometric measurements, glycemic parameters, and the expression of the SIRT1 gene, a protein enzyme involved in cellular metabolism. There was no significant difference between the effects of lifestyle modification and *N. sativa* on anthropometric measurements and most glycemic parameters. However, the lifestyle modification group exhibited significantly higher levels of homeostatic model assessment of β-cell function (HOMA-B), a mathematical model used to estimate β-cell function based on fasting glucose and insulin levels, and SIRT1 expression compared to *N. sativa* oil and metformin. Regarding lipid profile, *N. sativa* oil demonstrated improvements and significantly reduced TNF-α levels and Castelli risk index-I, an indicator of the risk of developing cardiovascular diseases, with higher values indicating an increased risk compared to other interventions (130). Besides, the administration of *N. sativa* oil to patients with type-2 diabetes could lessen the amounts of serum FBG, HbA1C, TC, TG, and LDL, as well as BMI, waist circumference, and systolic and diastolic blood pressure compared to the placebo group (131).

Likewise, *N. sativa* oil supplementation attenuated TNF-α transcription and blood levels, adiponectin receptor 1 (AdipoR1) expression, serum adiponectin gene expression, serum levels of PPAR-γ, and body weight of overweight/obese women (132).

The research consistently shows that *N. sativa* supplementation may benefit various populations, including individuals with type 2 diabetes, obese women, overweight individuals, and those with prediabetes or Hashimoto’s thyroiditis. *N. sativa* consumption had beneficial effects on anthropometric parameters such as BMI, body weight, waist circumference, and fat mass. It also had a favorable effect on glycemic indicators such as FBG, two-hour postprandial glucose levels, HbA1C, and insulin resistance. Furthermore, *N. sativa* supplementation was linked to improved lipid profiles, such as lower TC, TG, and LDL levels, as well as higher HDL. Furthermore, *N. sativa* showed anti-inflammatory properties, including lowering TNF-α levels and improving cardiovascular risk indicators.

*Phaseolus vulgaris*

*Phaseolus vulgaris *L. is a versatile legume from the Fabaceae family (133). Along with its culinary significance, *P. vulgaris* has been linked to potential health advantages. It contains a variety of bioactive components that have been researched for their medicinal potential. While studies are ongoing, *P. vulgaris* has shown promise in cardiovascular health (134), weight control (135), gastrointestinal health (136), and blood sugar regulation (137). 

The supplementation of *P. vulgaris* aqueous extract along with a carbohydrate-rich diet in overweight samples resulted in a decrease in BMI, adipose tissue thickness, body weight, fat mass, and waist/hip/thigh circumferences. But, it had no effect on lean body mass (138). Moreover, it has been demonstrated that consuming *P. vulgaris* snack bars, composed of beans and oats, without diet restriction could decrease serum levels of TG, glucose, and body weight in hypertriglyceridemic women in comparison to the control group. The proteome analysis performed in the study revealed that the bioactive compounds included in snack bars play a role in inhibiting monocyte recruitment and localized inflammatory responses. They also suppress pre-adipocyte development and adipogenesis, reduce hepatic beta-oxidation, and could regulate satiety (139) (Table 5).

A clinical trial investigated the effects of *P. vulgaris* aqueous extract, while following a calorie-restricted diet for weight management in individuals who were overweight or had moderate obesity. The results showed that the extract significantly reduced body weight, fat mass, BMI, waist circumference, hip circumference, and thigh circumference in a dose-dependent manner, particularly in the high dose group (140).

In summary, research has demonstrated that *P. vulgaris* supplementation might help in weight management in overweight people. It has been shown to lower BMI, adipose tissue thickness, body weight, fat mass, and waist/hip/thigh circumference. Notably, these results were accomplished without the requirement for calorie restriction. Additionally, the bioactive compounds of the extract have been shown to inhibit monocyte recruitment, limit pre-adipocyte formation and adipogenesis, reduce hepatic beta-oxidation, and regulate satiety. Generally, *P. vulgaris* shows its potential as a weight management supplement.

*Portulaca oleracea*

*Portulaca oleracea*, a succulent annual herbaceous plant from the Portulacaceae family, is commonly known as purslane. It is widely distributed in tropical and subtropical regions across the globe. Purslane has a long history of traditional medicinal use for various conditions, including liver and inflammatory disorders, gastrointestinal and skin diseases, as well as respiratory problems, fever, and bladder ulcers (11, 141). The World Health Organization has recognized purslane as a “Global Panacea” due to its extensive medicinal applications (142). Recent research has shed light on the therapeutic properties of *P. oleracea*, revealing its hepatoprotective (143), anti-oxidant (144), hypnotic (83), anti-inflammatory (145), anti-bacterial (146), and anti-fungal (147) effects, besides its anti-ulcerogenic properties (148). 

It has been observed that taking *P. oleracea* seeds reduced body weight, BMI, insulin, fasting and postprandial blood glucose, serum levels of LDL, TC, TG, GGT, AST, ALT, and direct and total bilirubin in obese individuals with type-2 diabetes in comparison to the metformin group. It also increased HDL and albumin (149) (Table 5). 

A clinical trial compared the effects of a weight loss diet versus a weight loss diet plus *P. oleracea* seeds on NAFLD patients. The obtained data indicated that following the intervention, both groups showed significant decreases in weight, waist circumference, and hip circumference; only the changes in hip circumference between the two groups were statistically significant. Just the *P. oleracea* group experienced a significant reduction in BMI. In both groups, there was a decrease in the average consumption of energy, protein, carbohydrates, and fat. The FBG differed considerably from the control group. *P. oleracea* consumption substantially reduced ALT and AST in the relevant group, but the control group did not experience any significant modifications. Liver steatosis decreased in both groups, but it was more pronounced in the *P. oleracea* group (150). Similarly, taking *P. oleracea* seeds along with low-fat yogurt resulted in lowered weight, BMI, serum TG, and systolic blood pressure in participants with type-2 diabetes compared to the control group that consumed low-fat yogurt (151).

The administration of aqueous ethanolic extract of aerial parts of *P. oleracea* in NAFLD patients resulted in decreased NF-κB p65 nuclear activity, weight, waist circumference, and BMI compared to the baseline (152). Likewise, it has been illustrated that the administration of *P. oleracea* freeze-dried juice to individuals with a BMI >25.0 kg/m² attenuated their BMI and appetite versus the placebo group (153).

The findings from various studies suggest that the consumption of *P. oleracea* seeds or its extracts can have positive effects on weight loss, BMI, blood glucose levels, lipid profiles, liver function, and other health parameters. One of the strengths of the studies mentioned is that they examined different populations, including obese individuals with type-2 diabetes, NAFLD patients, and individuals with a BMI >25.0 kg/m², increasing the generalizability of the findings to diverse populations. 

Despite the promising findings, there are some limitations to consider. Firstly, the sample sizes in these studies were relatively small, which may limit the generalizability of the results. Additionally, the duration of the interventions varied among the studies, making it challenging to draw conclusions about long-term effects. Furthermore, most of the studies focused on specific populations, such as obese individuals with type-2 diabetes or NAFLD patients, limiting the applicability of the findings to the general population. Future research should aim to address these limitations by conducting larger-scale, long-term studies involving diverse populations. 

*Salvia rosmarinus*

Rosemary, scientifically referred to as *Salvia rosmarinus* Spenn, is a perennial shrub that retains its green foliage throughout the year. It belongs to the Lamiaceae family. Initially found in the Mediterranean area, rosemary is now grown globally because of its role as a natural food preservative and enhancer of taste (154). Additionally, for many centuries, rosemary has been utilized as a traditional medicinal resource (155). Physio-pharmacological investigation illustrated its anti-inflammatory (156, 157), anti-oxidant, anti-apoptotic (158, 159), anti-nociceptive (160), neuroprotective (161, 162), anti-rheumatic (163), anti-dote (164), cardioprotective (165, 166) properties. 

The effect of rosemary leaf powder, combined with a weight loss diet and physical activity recommendations, was investigated in NAFLD patients. Participants were divided into two groups: one received rosemary leaf powder, while the other received a placebo in the form of starch powder. After comparing initial measures, both the rosemary and placebo groups showed significant changes in different biomarkers and anthropometric indices. These improvements included ALT, AST, ALP, GGT, FBG, fasting insulin, insulin resistance, TC, TG, LDL, and weight, BMI, and waist circumference. After 8 weeks, there were no significant differences between the rosemary and placebo groups in the aforementioned characteristics, except for the assessment of β-cell dysfunction using the homeostasis model (Table 5) (167). 

The effects of administering a dry *S. rosmarinus *extract along with aesthetic radiofrequency therapy were investigated in a clinical trial on anthropometric measurements in healthy women. The results showed that there were no notable modifications to the assessed parameters as a result of the aesthetic radiofrequency therapy. On the other hand, the application of *S. rosmarinus *resulted in substantial alterations, including a linear, dose-dependent increase in skinfold thickness for all tested doses. Furthermore, a decrease in abdomen circumference was observed following administration of a 500 mg dose (168). 

Hence, although *S. rosmarinus*, when combined with a weight loss diet and physical activity recommendations, did not show significant alterations compared to the placebo group in the NAFLD study, it demonstrated significant improvements when compared to aesthetic radiofrequency therapy in the second study. This suggests that *S. rosmarinus *may be a promising option for individuals who are not willing to consider dietary changes or engage in exercise. The strengths of both studies include their respective designs and comprehensive assessments. However, limitations such as short duration, sample size, and specific contexts should be considered. 

*Zataria multiflora*

*Zataria multi*flora Boiss belongs to the Lamiaceae family and has small, narrow, and elliptical leaves. It is native to central and southern Iran, Pakistan, Oman, the West Himalayas, and Afghanistan, where it is known as Avishan Shirazi in Persian (169, 170). The plant has numerous advantages, including anti-oxidant, neuroprotective (171), anti-tussive, anti-microbial, anti-asthmatic, analgesic, and anti-inflammatory properties (172). Some studies have highlighted the potential anti-obesity properties of *Z. multiflora*, which will be discussed in the following section.

The administration of *Z. multiflora *to overweight people decreased waist circumference, hip circumference, and insulin resistance in comparison to the placebo group. However, it had no effect on BMI (173). Moreover, the consumption of *Z. multiflora*, along with exercise training, attenuated serum levels of angiopoietin-like protein 8 (ANGPTL8), which regulates lipid metabolism and glucose homeostasis. It also reduced the levels of ICAM-1, facilitating cell-to-cell adhesion and immune response by mediating interactions between cells and leukocytes, as well as body fat mass in sedentary overweight and obese men (174) (Table 6).

To summarize, *Z. multiflora* supplementation reduced waist circumference, hip circumference, insulin resistance, serum levels of ANGPTL8, ICAM-1, and body fat mass in overweight adults, sedentary overweight, and obese men. 

*Zingiber officinale*

*Zingiber officinale *Roscoe, a member of the Zingiberaceae family, is a popular spice and medicinal plant used globally. Scientific data supports its several pharmacological actions, which include anti-oxidant and anti-inflammatory properties (175), fat and glucose reduction (176), anti-emetic properties (177), and anti-cancer properties (178). Recent research has also examined the anti-obesity and weight-loss properties of ginger and its constituents, and some encouraging findings have been reported (179-181).

The consumption of *Z. officinale* rhizomes powder in obese women led to attenuated body weight, BMI, waist and hip circumferences, dietary intake, and appetite score compared to the placebo group. Though, it did not affect macronutrients intake, total energy, and body composition (182). Similarly, it has been found that *Z. officinale* rhizomes powder lessened BMI and HOMA-IR, and increased quantitative insulin sensitivity check index in obese women (183).

It has been shown that the administration of *Z. officinale* powder in individuals newly diagnosed with type-2 diabetes enhanced HDL, β-cell function index, and insulin sensitivity index. It also lowered BMI, FBG, two-hour postprandial glucose levels, HbA1C, TC, LDL, TG, fasting insulin levels, and HOMA2-IR compared to the placebo group (184). Furthermore, taking steamed ethanol extract of *Z. officinale* resulted in decreased body weight, BMI, and body fat levels in healthy obese people (Table 6) (185).

The data of another clinical study revealed that receiving *Z. officinale* extract had no substantial effect on resting energy expenditure and body composition in females with high body adiposity (186).

The supplementation of *Z. officinale* powder to NAFLD patients caused no considerable alteration in body weight. However, it reduced ALT, TC, LDL, FBG, HOMA, hs-CRP, and fetuin-A, a chief carrier protein of free fatty acids in blood circulation, in comparison to the placebo group (187). It has also been shown that the administration of *Z. officinale* powder to participants with type-2 diabetes who have NAFLD increased HDL and decreased BMI, waist and hip circumferences, amounts of liver transaminase, serum insulin, and HOMA-IR compared to the baseline (188).

In summary, *Z. officinale* powder has shown positive effects on weight management, insulin sensitivity, and lipid profiles in various health conditions, including obesity, type-2 diabetes, and NAFLD. While the studies mentioned present some contradictory findings regarding the effects of *Z. officinale* on certain parameters, it is important to consider that research in this field is still evolving, and further investigations are needed to fully understand the potential benefits and mechanisms of action. The contradictory results may be attributed to several factors, including variations in study design, sample sizes, duration of intervention, dosage, and the specific population under study. Additionally, variations in the formulation and preparation of ginger extracts or powders used in different studies could contribute to the differences observed.

To address these contradictions and gain a clearer understanding of the effects of *Z. officinale*, future research should focus on conducting well-designed, large-scale clinical trials with standardized protocols. This would help to establish optimal dosages, treatment durations, and identify specific subpopulations that may benefit the most from *Z. officinale* supplementation. 

## Future developments

Although this comprehensive review sheds light on the current state of research on herbal medicines for weight loss, there are still a number of topics that demand more study. Future research projects should primarily focus on in-depth mechanistic studies. These studies should aim to clarify the biological pathways and molecular targets through which herbal medicines influence weight loss, enhancing our understanding and application of these treatments. By elucidating the specific actions of herbal compounds at the cellular and molecular levels, researchers can identify potential synergies with conventional therapies, optimize dosages, and modify treatments to individual patient needs. Furthermore, a deeper understanding of the mechanisms involved can help in addressing safety concerns, potential side effects, and interactions with other medications. Also, this knowledge will not only improve the efficacy of herbal treatments but also facilitate their integration into ordinary weight management strategies, providing a more complete approach to obesity treatment. To ensure consistent efficacy and potency, standardized procedures for the extraction, formulation, and production of herbal medicines must be established. Furthermore, it is essential to develop rigorous quality control protocols to evaluate the existence of contaminants and substances in herbal products to guarantee their safety and effectiveness. Long-term research is also necessary to assess the safety or efficacy of herbal medications for weight loss over prolonged periods of time. Furthermore, it is crucial to do research on a variety of populations, including those with particular medical conditions like diabetes, cardiovascular diseases, or metabolic disorders, as well as individuals from various age groups and ethnic backgrounds. Additionally, it would be beneficial to conduct research regarding the potential advantages of combining herbal medicines with traditional weight loss methods such as dietary changes, exercise routines, and behavioral therapy. 

## Conclusion

This comprehensive review of clinical trials examining the use of herbal medicines (*A. sativum*, *C. sinensis*, *C. verum*, *C. sativus*, *C. longa*, *G. mangostana,* *N. sativa*, *P. vulgaris*, *P. oleracea*, *S. rosmarinus*, *Z. multiflora*, and *Z. officinale*) for weight loss sheds light on their potential as adjunctive therapies in weight management. The findings indicate that certain herbal medicines exhibit promising effects on weight reduction and metabolic parameters, contributing to overall well-being.

**Figure 3:**
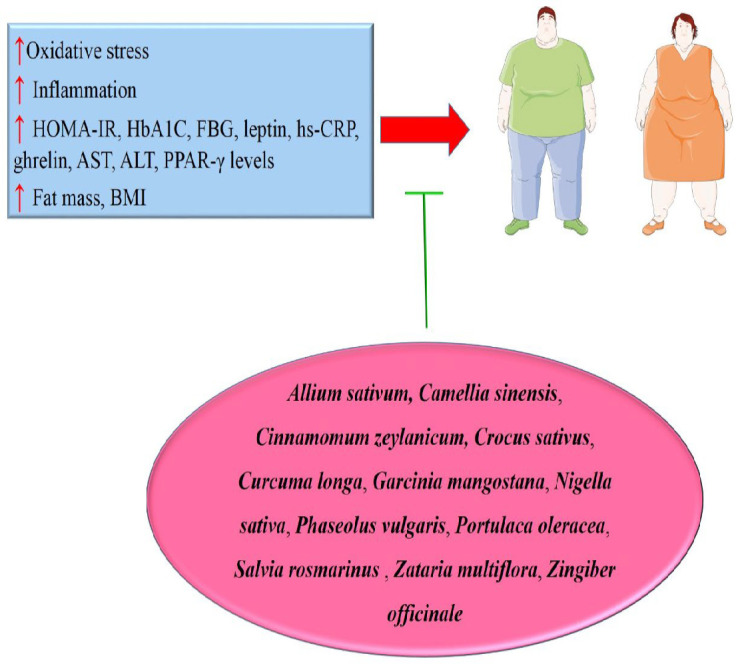
Effects of herbal medicines on obesity management (Images from https://smart.servier.com)

The reviewed evidence suggests that these herbal medicines may facilitate weight loss through various mechanisms. Specifically, they have been shown to decrease FBG and HOMA-IR and regulate insulin levels, which are critical factors in metabolic health. Additionally, these herbal extracts can reduce MDA levels, indicating a potential anti-oxidant effect. Moreover, the herbal interventions have been associated with increased levels of CAT and GSH, which play essential roles in oxidative stress reduction (Figure 3). The modulation of appetite and satiety is also evident, as these herbal medicines can influence ghrelin levels and reduce dietary and energy intake, leading to decreased fat mass values, waist circumference, and BMI.

The impact on lipid profiles is noteworthy, with reductions in the LDL/HDL ratio, TC, TG, and serum levels of TNF-α, hs-CRP, and insulin. Furthermore, these herbal extracts have been linked to decreased transcription and blood levels of TNF-α, enhanced expression of adiponectin and PPAR-γ, and reduced NF-κB p65 nuclear activity, all of which are crucial for regulating inflammation and metabolic processes.

While herbal medicines offer potential benefits, it is essential to approach their use with caution. The observed side effects, such as muscle loss induced by curcumin in advanced pancreatic cancer, highlight the importance of carefully considering the safety profiles of herbal interventions. Rigorous research, including long-term studies with larger sample sizes, is necessary to comprehensively evaluate the safety and long-term effects of consuming herbal medicines for weight loss. Moreover, the standardization of herbal preparations and quality control measures are vital to ensure the consistency, potency, and purity of herbal products. Adherence to evidence-based guidelines and collaboration between healthcare professionals, researchers, and traditional medicine practitioners are crucial for integrating herbal medicines into mainstream weight management strategies.

**Table 1:** Clinical evidence of anti-obesity effects of* Allium sativum* and *Camellia sinensis* in human studies

Compounds	Participants	Doses/duration	Results	Ref.
*Allium* * sativum *powder	110 NAFLD patients	400 mg, 15 weeks	No changes in lean body mass and total body water ↓Body weight and body fat mass	(49)
*A. sativum *powder	180 postmenopausal women	500 mg, twice daily, 12 weeks	↑ Muscle mass ↓ Body fat and visceral fat levels	(50)
*A. sativum *powder	90 NAFLD patients	400 mg, 12 weeks	↑ Skeletal muscle mass, serum concentration of SOD level, and total anti-oxidant capacity ↓ Waist circumference, body fat percent, FBG, insulin, HOMA-IR, MDA	(51)
*A. sativum *extract (Allium-S tablet)	32 obese women	400 mg, 8 weeks	↑*Faecalibacterium* and *Bifidobacterium* abundance ↓BMI, fasting insulin level, HOMA-IR, *Akkermansia* frequency	(52)
*A. sativum *pill	80 samples with polycystic ovary syndrome	800 mg, 8 weeks	↑CAT and GSH levels ↓ Weight, BMI, waist circumference	(53)
Monoselect Camellia® (MonCam)	50 obese individuals	90 days	↑ Weight loss ↓BMI, waistline (just in males), leptin	(62)
*C. sinensis* with high catechin	182 moderately overweight Chinese participants	458, 468, and 886 mg catechins, 90 days	↓ Intra-abdominal fat, waist circumference, body weight, total body fat	(63)
Epigallocatechin-3-O-gallate	83 obese premenopausal Caucasian women with BMI of 30-40 kg/m^2^	300 mg, 12 weeks	-No significant effect on body weight, fat mass, energy and fat metabolism, HOMA-IR, TC, LDL, TG, liver function markers	(64)
*C. sinensis*	60 Caucasian	>0.56 g/d epigallocatechin gallate + 0.28–0.45 g/d caffeine	-No significant effect on fecal energy content, fecal fat content, resting energy expenditure, respiratory quotient, and body composition	(65)
*C. sinensis* leaves water extract	102 women with central obesity	500 mg, three times daily, 12 weeks	↑Adiponectin levels ↓Weight, BMI, waist circumference, TC, LDL, ghrelin levels	(66)
*C. sinensis*	84 healthy people with BMI of 24-35 kg/m^2^	a cup 30 min after breakfast and lunch, 12 weeks	↓Weight, BMI	(67)
Epigallocatechin-3-O-gallate	60 healthy Japanese	146 mg, 12 weeks	-Inhibited weight gain ↓BMI, TG, body fat percentage, visceral fat level, body weight, body fat percentage, blood LDL/HDL ratio	(54)

BMI: Body mass index; CAT: Catalase; FBG: Fasting blood glucose; GSH: Glutathione; HDL: High-density lipoprotein; HOMA-IR: Homeostatic model assessment of insulin resistance; LDL: Low-density lipoprotein; MDA: Malondialdehyde; NAFLD: Nonalcoholic fatty liver disease; SOD: Superoxide dismutase; TC: Total cholesterol; TG: Triglycerides.

**Table 2:** Clinical evidence of anti-obesity effects of *Cinnamomum verum* and *Crocus sativus* in human studies

Compounds	Participants	Doses/Duration	Results	Ref.
*Cinnamomum* * verum*	37 people with type-2 diabetes	3 g, 8 weeks	↓ FBG, HbA1C, TG, weight, BMI, and body fat mass	(70)
*C. verum*	81 people with type-2 diabetes	1000 and 2000 mg, 3 months	↓Weight, waist circumference, and BMI	(69)
Cinnamon	84 overweight/obese participants with polycystic ovary syndrome	500 mg, three times daily, 8 weeks	↑HDL ↓FBG, HOMA-IR, TC, LDL, weight, TG, BMI	(71)
*C. verum*	84 obese individuals with type-2 diabetes	4 and 8 g, 10 weeks	↑Ghrelin secretion ↓BMI, waist circumference, levels of blood glucose, insulin	(72)
*C. verum*	83 women with polycystic ovary syndrome	500 mg, three times daily	↓ Weight, BMI, HOMA-IR, testosterone levels	(73)
*C. verum* bark powder	50 migraine patients	600 mg, 2 months	-Prevented the increase in body weight and BMI ↓ Hip circumference and headache daily result	(74)
*C. verum* blume and inulin	58 patients with metabolic syndrome and type-2 diabetes or impaired glucose tolerance	4 g, 4 months	↓ Weight, BMI, serum insulin, and HOMA index, lipemic pattern, TC, TG, LDL	(75)
Satiereal (Inoreal Ltd, Plerin, France), extract of *Crocus sativus* stigma	60 healthy, mildly overweight women	176.5 mg extract, 8 weeks	↑Weight loss ↓Mean snacking frequency	(90)
*C.* *sativus* aqueous extract and crocin	84 patients with coronary artery disease	30 mg, 8 weeks	↓ Fat mass values, waist circumference, BMI, dietary and energy intake, appetite	(91)
Crocin	48 patients with metabolic syndrome	100 mg, 6 weeks	-No significant changes in anthropometric measures	(92)
*C.* *sativus*	75 overweight/obese prediabetic individuals	15 mg, 8 weeks	-No significant changes in anthropometric measures	(93)
*C.* *sativus*	80 patients with type-2 diabetes	100 mg, 12 weeks	↓Waist circumference -No significant changes in other anthropometric measures	(94)
*C.* *sativus*	76 NAFLD patients	100 mg, 12 weeks	-No significant effects on weight, BMI, and visceral fat level ↓ hs-CRP and leptin	(95)
*C.* *sativus*	73 individuals with BMI ≥ 25 and depression	15 mg twice daily, 12 weeks	↑Weight loss and depression scores	(96)
*C.* *sativus*	80 participants with mild to moderate ulcerative colitis	100 mg, 8 weeks	-No significant changes in anthropometric measures	(97)
*C.* *sativus* stigma powder	74 obese prediabetic adolescents	60 mg, 12 weeks	↓ BMI, waist circumference, weight z-score, BMI z-score, TG, HDL	(76)

BMI: Body mass index; FBG: Fasting blood glucose; HbA1C: glycosylated hemoglobin; HDL: High-density lipoprotein cholesterol; HOMA-IR: Homeostatic model assessment of insulin resistance; hs-CRP: High-sensitivity C-reactive protein; LDL: Low-density lipoprotein; TC: Total cholesterol; TG: Triglycerides.

**Table 3:** Clinical evidence of anti-obesity effects of *Curcuma longa* and *Garcinia mangostana* in human studies

Compounds	Participants	Doses/Duration	Results	Ref.
Curcumin	30 obese individuals	1 g, 30 days	-No significant effects on weight and BMI ↓Anxiety scores	(101)
Curcumin	66 individuals with advanced pancreatic cancer	2 months	↑ Muscle loss ↓ Body weight and subcutaneous fat	(102)
Curcumin	80 NAFLD patients	70 mg, 8 weeks	↓ Liver fat content, BMI, TC, LDL, TG, AST, ALT, serum glucose, HbA1C	(103)
Curcumin	53 overweight samples with type-2 diabetes	1500 mg, 10 weeks	↓Weight, BMI, waist circumference, and FBG	(104)
*Curcuma * *longa* powdered rhizome	42 women with hyperlipidemia and type-2 diabetes	2,100 mg, 8 weeks	↓ Body weight, BMI, body fat percentage, and waist-to-hip ratio	(105)
*C.* *longa* supercritical carbon dioxide extract	90, overweight 50 to 69 years healthy subjects	2 capsules, 12 weeks	↑ Mental health ↓Body weight, BMI, complement component 3, serum CRP	(106)
Curcumin (Curcumin C3 Complex®)	35 Brazilian women with high waist circumferences	500 mg, 90 days	↓ Body mass, FBG, TG, and TC	(107)
Calebin A	94 obese patients	25 mg, 90 days	↑ HDL ↓Body weight, BMI, waist circumference, LDL, TG, CRP, leptin	(108)
XanGo Juice™	122 individuals with BMI ≥ 30 and ≤ 45	3, 6, or 9 oz twice daily, 8 weeks	↓hs-CRP and BMI	(115)
*G. mangostana*- based drink	60 healthy people	245 mL/day, 30 days	-No significant changes in the weight and BMI ↑Blood anti-oxidant capacity ↓CRP	(116)
*G. mangostana* supplement	20 obese females with BMI ≥ 30 kg/m^2^ and body weight less than 135 kg	400 mg/day, 26 weeks	↑Weight loss	(117)

ALT: Alanine aminotransferase; AST: Aspartate aminotransferase; BMI: Body mass index; CRP: C-reactive protein; FBG: Fasting blood glucose; HbA1C: glycosylated hemoglobin; HDL: High-density lipoprotein cholesterol; hs-CRP: High-sensitivity C-reactive protein; LDL: Low-density lipoprotein; TC: Total cholesterol TG: Triglycerides

**Table 4:** Clinical evidence of anti-obesity effects of *Nigella sativa* in human studies

Compounds	Participants	Doses/Duration	Results	Ref.
*Nigella* * sativa *oil	70 type-2 diabetic people	2.5 ml, 3 months	↓BMI, HbA1C, FBG, 2 hours postprandial glucose levels	(119)
*N. sativa *crushed seeds	20 sedentary overweight women	2 g, 8 weeks	↑HDL ↓TC, TG, LDL, BMI	(120)
*N. sativa *oil	49 obese women	3 g, 8 weeks	↑SOD levels of red blood cells ↓ Weight	(121)
*N. sativa *oil	84 obese women with BMI of 30-35 kg/m^2^	3 g, 8 weeks	↓ Weight, waist circumference, TG, LDL	(122)
*N. sativa *oil	72 individuals with type-2 diabetes	3 g, 12 weeks	↓BMI, body weight compared to the baseline, HbA1C, FBG, TG, LDL compared to the placebo	(123)
*N. sativa *oil	90 obese women with BMI of 30-34.9 kg/m^2^	3 g, 8 weeks	-No significant effects on BMI and serum IL-6 ↓ Weight, serum levels of TNF-α, hs-CRP	(124)
*N. sativa *oil	50 obese women	3 g, 8 weeks	↑Adiponectin levels ↓Body fat mass and insulin amounts	(125)
*N. sativa *oil	100 obese women	8 weeks	↓Weight, insulin resistance, insulin concentrations	(126)
*N. sativa *powdered seeds	40 samples with Hashimoto’s thyroiditis	2 g, 8 weeks	↓BMI, body weight, LDL, TG	(127)
*N. sativa *oil	44 newly diagnosed type-2 diabetes individuals	1350 mg, 3 months	↓Weight, waist circumference, BMI	(128)
*N. sativa *oil	45 obese or overweight healthy women	2000 mg, two 8-week	↓BMI, body weight, waist circumference, body fat mass, body fat percent, and visceral fat area	(129)
*N. sativa *oil	117 obese prediabetic samples	450 mg, 6 months	↓Weight, BMI, TNF-α levels, Castelli risk index-I	(130)
*N. sativa *oil	43 patients with type-2 diabetes	500 mg, 8 weeks	↓ FBG, HbA1C, TC, TG, LDL, BMI, waist circumference, systolic and diastolic blood pressure	(131)
*N. sativa *oil	46 overweight/obese women	2000 mg, two 8-week	↓TNF-α transcription and blood levels, AdipoR1 expression, serum adiponectin gene expression and serum levels of PPAR-γ, body weight	(132)

AdipoR1: Adiponectin receptor 1; BMI: Body mass index; CRP: C-reactive protein; FBG: Fasting blood glucose; HbA1C: glycosylated hemoglobin; HDL: High-density lipoprotein; hs-CRP: High-sensitivity C-reactive protein; LDL: Low-density lipoprotein; PPAR-γ: Peroxisome proliferator-activated receptor-gamma; TC: Total cholesterol TG: Triglycerides; TNF-α: Tumor necrosis factor-alpha.

**Table 5:** Clinical evidence of anti-obesity effects of *Phaseolus vulgaris*, *Portulaca oleracea*, and *Salvia rosmarinus* in human studies

Compounds	Participants	Doses/Duration	Results	Ref.
*Phaseolus* vulgaris aqueous extract	60 overweight samples	445 mg, 30 days	-No effect on lean body mass ↓ BMI, adipose tissue thickness, body weight, fat mass, waist,/hip/ thigh circumferences	(138)
*P. *vulgaris snack bar	26 hypertriglyceridemic women	50 g, 8 weeks	↓Serum levels of TG, glucose, body weight	(139)
*P. *vulgaris aqueous extract	81 individuals with overweight and moderate obesity	700 and 1000 mg, 12 weeks	↓ BMI, body weight, fat mass, waist, hip	(140)
*Portulaca* * oleracea* seeds	30 obese participants with type-2 diabetes	5 g, twice daily, 8 weeks	↑HDL, albumin ↓Body weight, BMI, insulin, fasting, and postprandial blood glucose, Serum levels of LDL, TC, TG, GGT, AST, ALT, direct and total bilirubin	(149)
*P. oleracea* seeds	60 people with NAFLD	10 g/day, 8 weeks	↓Weight, BMI, hip and waist-hip circumference, average intake of carbohydrate, energy, fat, and protein, ALT, AST, liver steatosis	(150)
*P. oleracea* seeds	48 participants with type-2 diabetes	10 g/day, 5 weeks	↓Weight, BMI, serum TG, systolic blood pressure	(151)
Aqueous ethanolic extract of aerial parts of *P. oleracea*	71 individuals with NAFLD	300 mg, 12 weeks	↓NF-κB p65 nuclear activity, weight, waist circumference, BMI	(152)
*P. oleracea* freeze-dried juice	70 healthy individuals with a BMI >25.0 kg/m²	8 weeks	↓BMI and appetite	(153)
*Salvia rosmarinus* leaf powder	110 NAFLD patients	4 g/day, 8 weeks	-No significant changes between the rosemary and placebo groups in measured variables except homeostasis model assessment of β-cell dysfunction	(167)
*S.* * rosmarinus* dry extract	32 healthy woman	100, 500, and 1000 mg/day, 4 weeks	↑ Skinfolds ↓Abdominal circumference	(168)

ALT: Alanine aminotransferase; AST: Aspartate aminotransferase; BMI: Body mass index; CRP: C-reactive protein; FBG: Fasting blood glucose; HbA1C: glycosylated hemoglobin; HDL: High-density lipoprotein; hs-CRP: High-sensitivity C-reactive protein; GGT: Gamma-glutamyl transferase; LDL: Low-density lipoprotein; NF-κB: Nuclear factor-kappa B; TC: Total cholesterol TG: Triglycerides

**Table 6:** Clinical evidence of anti-obesity effects of *Zataria multiflora *and *Zingiber officinale* in human studies

Compounds	Participants	Doses/duration	Results	Ref.
*Zataria* * multiflora*	108, overweight people	0.75 and 1.5 g, 12 weeks	-No significant change in BMI ↓Waist circumference, hip circumference, insulin resistance	(173)
*Z. multiflora*	40 sedentary, overweight, and obese men	500 mg, 8 weeks	↓Serum levels of ANGPTL8, ICAM-1, body fat mass	(174)
*Zingiber* * officinale* rhizomes powder	80 obese women	2 g, 12 weeks	-No effect on macronutrients intake, total energy, body composition ↓ Body weight, BMI, waist and hip circumferences, dietary intake, appetite score	(182)
*Z. officinale* rhizomes powder	80 obese women	2 g, 12 weeks	↑Quantitative insulin sensitivity check index ↓BMI and HOMA-IR	(183)
*Z. officinale* powder	80 individuals newly diagnosed with type-2 diabetes	1.8 g, 8 weeks	↑ HDL, β-cell function index, insulin sensitivity index ↓BMI, FBG, two hours postprandial glucose levels, HbA1C, TC, LDL, TG, fasting insulin levels, HOMA2-IR	(184)
Steamed* Z. officinale* ethanolic extract	80 healthy obese people	200 mg, 12 weeks	↓ Body weight, BMI, body fat level	(185)
*Z. officinale* extract	66 female with high body adiposity	600 mg, 3 months	No significant effect on resting energy expenditure and body composition	(186)
*Z. officinale* powder	46 samples with NAFLD	1.5 g, 12 weeks	No significant effect on body weight ↓ALT, TC, LDL, FBG, HOMA, hs-CRP, and fetuin-A	(187)
*Z. officinale* powder	76 participants with type-2 diabetes who have NAFLD	1000 mg, twice daily, 3 months	↑HDL ↓ BMI, waist and hip circumferences, amounts of liver transaminase, serum insulin, and HOMA-IR	(188)

ALT: Alanine aminotransferase; ANGPTL8: Angiopoietin-like protein 8; BMI: Body mass index; CRP: C-reactive protein; FBG: Fasting blood glucose; HbA1C: glycosylated hemoglobin; HDL: High-density lipoprotein; hs-CRP: High-sensitivity C-reactive protein; ICAM-1: Intercellular adhesion molecule-1; LDL: Low-density lipoprotein; TC: Total cholesterol TG: Triglycerides

## Data Availability

Data sharing is not applicable to this article as no datasets were generated or analyzed during the current study.

## Declaration

During the preparation of this work the author(s) used ChatGPT and Quillbot in order to rephrase to reduce plagiarism and improve the language and grammar. After using this tool/service, the authors reviewed and edited the content as needed and take full responsibility for the content of the publication.
